# Pelvic Bone Hydatidosis: A Dangerous Crippling Disease

**DOI:** 10.7759/cureus.4465

**Published:** 2019-04-16

**Authors:** Faisal Inayat, Raza E Rana, Shoaib Azam, Rizwan Ahmad, Soban Ahmad

**Affiliations:** 1 Internal Medicine, Allama Iqbal Medical College, Lahore, PAK; 2 Orthopaedics, King Edward Medical University, Lahore, PAK; 3 Internal Medicine, Lahore General Hospital, Lahore, PAK; 4 Emergency Medicine, Hamad Medical Corporation, Doha, QAT

**Keywords:** hydatid disease, pelvic bone, echinococcus, epidemiology, clinical presentation, diagnosis, management, curettage, clinical outcomes

## Abstract

Hydatid disease is one of the most complicated and devastating conditions caused by *Echinococcus granulosus*. This globally distributed disease continues to be an important public health concern in many low- and middle-income countries. The liver and the lungs are the most frequently involved sites, but virtually any organ system can be affected. Osseous hydatidosis is relatively less common, but it is extremely debilitating and difficult to manage due to frequent recurrences. Patients often demonstrate a delayed clinical presentation as bone involvement is predominantly a silent and slowly progressive disease with a long latent period. Radiological investigations play an important role in the diagnosis. Although standard therapeutic guidelines are not available, the treatment of choice is a combination of chemotherapy and surgery. Clinicians should perform a lifelong follow-up for early detection of potential recurrence and sequels. This paper aims to highlight hydatid disease of the pelvic bone as an important differential diagnosis of tubercular hip arthritis, especially in areas with high echinococcosis prevalence.

## Introduction

Hydatid disease is a complex zoonosis most commonly caused by *Echinococcus granulosus.* Although the two frequent locations of the cystic disease are liver and lung, it may also appear in other parts of the body [1]. Osseous hydatidosis is relatively rare, with an estimated incidence up to 4% of all cases [2]. Hydatid disease of bone mainly damages trabeculae, but cortices can be eroded along with the involvement of adjacent soft tissues [3]. With regard to the diagnosis, clinical history, laboratory evaluation such as serological detection of specific immunoglobulin G (IgG) antibodies, radiologic investigations, and histopathologic analysis of the biopsy specimen are of paramount importance [4-5]. Bone disease is particularly difficult to diagnose, given the vague symptomatology and typically delayed clinical presentation [6]. The appropriate management includes various combinations of surgery, percutaneous treatment, and antihelminthic chemotherapy [7].

We chronicle here an interesting case of hydatid disease involving the pelvic bone, with no evidence of primary lesion in the liver. The patient initially presented with mild pain but it gradually worsened. The diagnosis was established on the standard set of investigations. In addition to chemotherapy, he was managed with an uneventful surgical resection of the involved portions of the pelvic bone. This paper prompts clinicians to maintain a high index of suspicion for bone hydatidosis, especially in patients presenting with nonspecific skeletal symptoms. Furthermore, it re-emphasizes the importance of thorough evaluation in these cases. In patients with a late diagnosis of osseous hydatidosis, recurrence of the symptoms has frequently been noted. Therefore, appropriate treatment early in the course of the disease is imperative to minimize morbidity and mortality.

## Case presentation

A 23-year-old male presented to our medical center with pain in the right lower back for three years. The pain was initially mild, gradually increased in intensity and aggravated on walking. Fever, anorexia, cough, and weight loss were not present. The patient was a farmer who occasionally went for cattle herding. His medical history was negative for trauma, recent infection, and prior medical conditions. Physical examination revealed a moderately tender swelling at the right lower back. The gait of the patient was antalgic but rest of the motor examination was unremarkable.

Investigations

**Figure 1:**
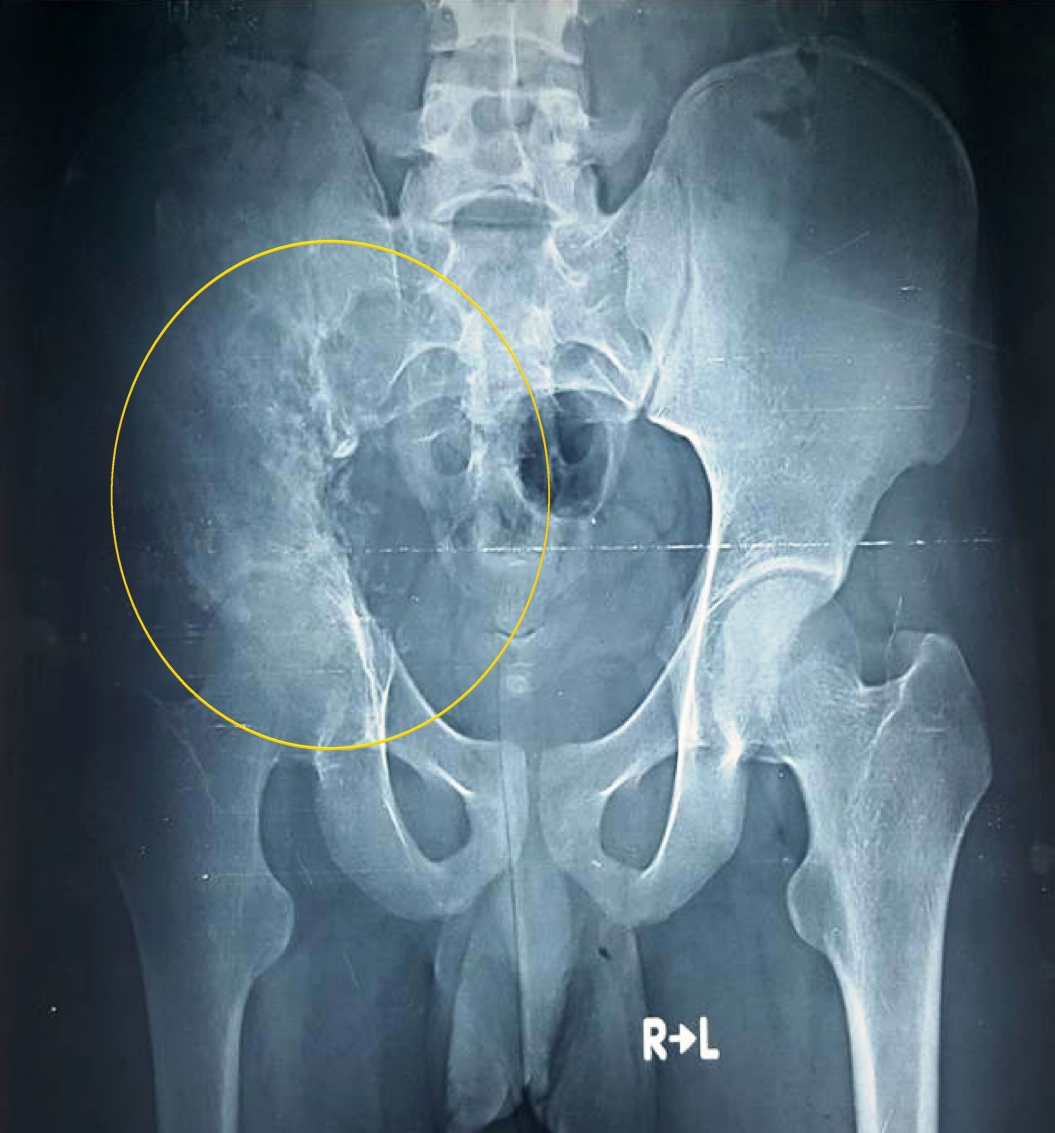
Pelvic radiograph showing osteolytic lesions in the right ilium, ischium, and sacroiliac and hip joints (anteroposterior view; circle)

Laboratory studies were significant for white cell count 19.6×10^9^/L (normal, 4.5-11.0×10^9^/L) and C-reactive protein (CRP) 269 mg/dL (normal, <3.0 mg/L). Plain radiograph of the pelvis revealed osteolytic lesions in the right ilium and ischium, with extensions to the sacroiliac and hip joints (Figure 1).

**Figure 2:**
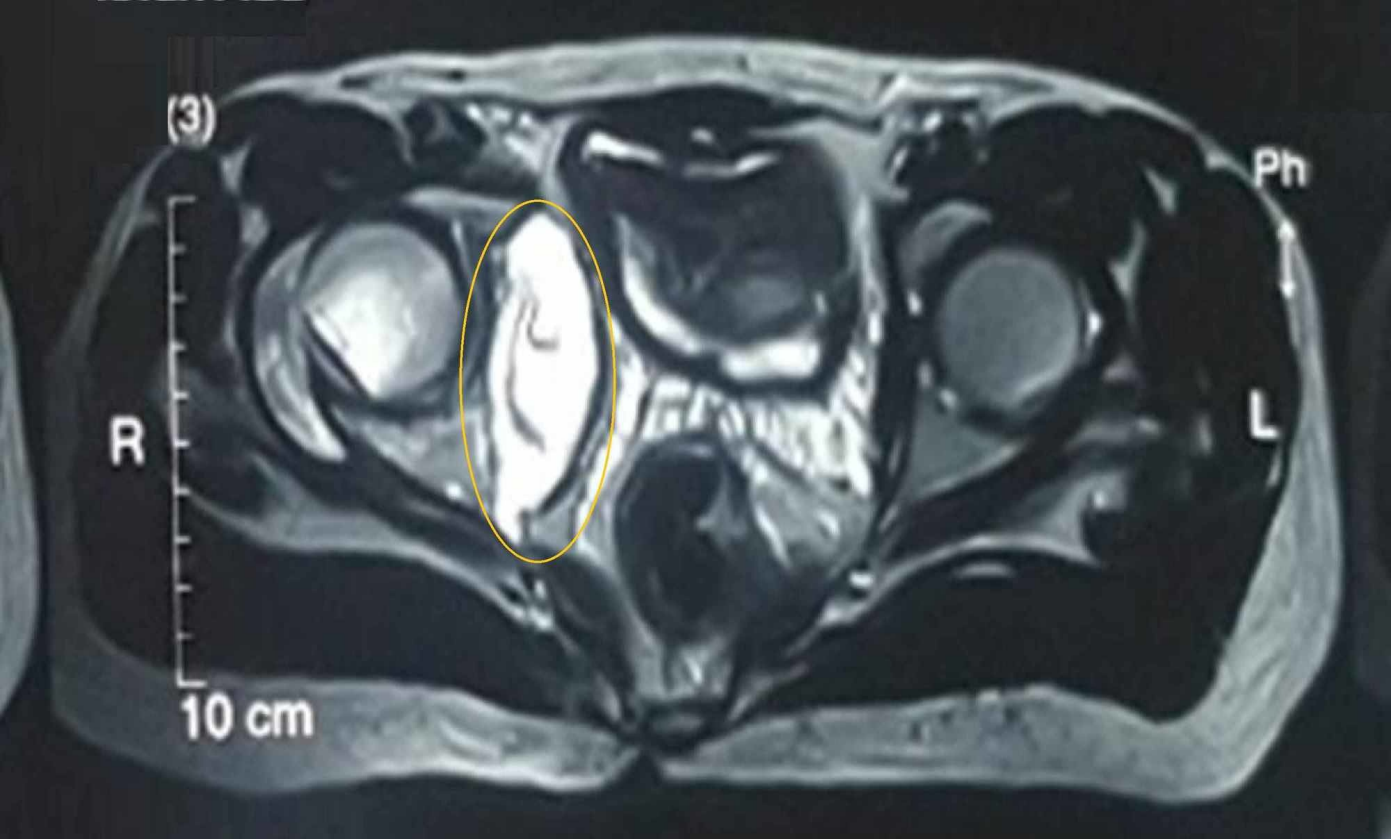
Magnetic resonance imaging of pelvis showing a homogeneous hyperintense lesion on T2-weighted image in the right iliac blade, right sacroiliac, and hip joints A hypointense rim at the periphery of the cystic lesion with the “water-lily sign” was noted (circle).

Magnetic resonance imaging (MRI) of the pelvis showed a large heterogeneous signal intensity mass, centered around the right iliac blade, extending to both the iliac and gluteal sides of the bone and was associated with an extensive bony destruction of the right sacroiliac joint and ischium with a cystic component in the right iliopsoas muscle (Figure 2).

**Figure 3:**
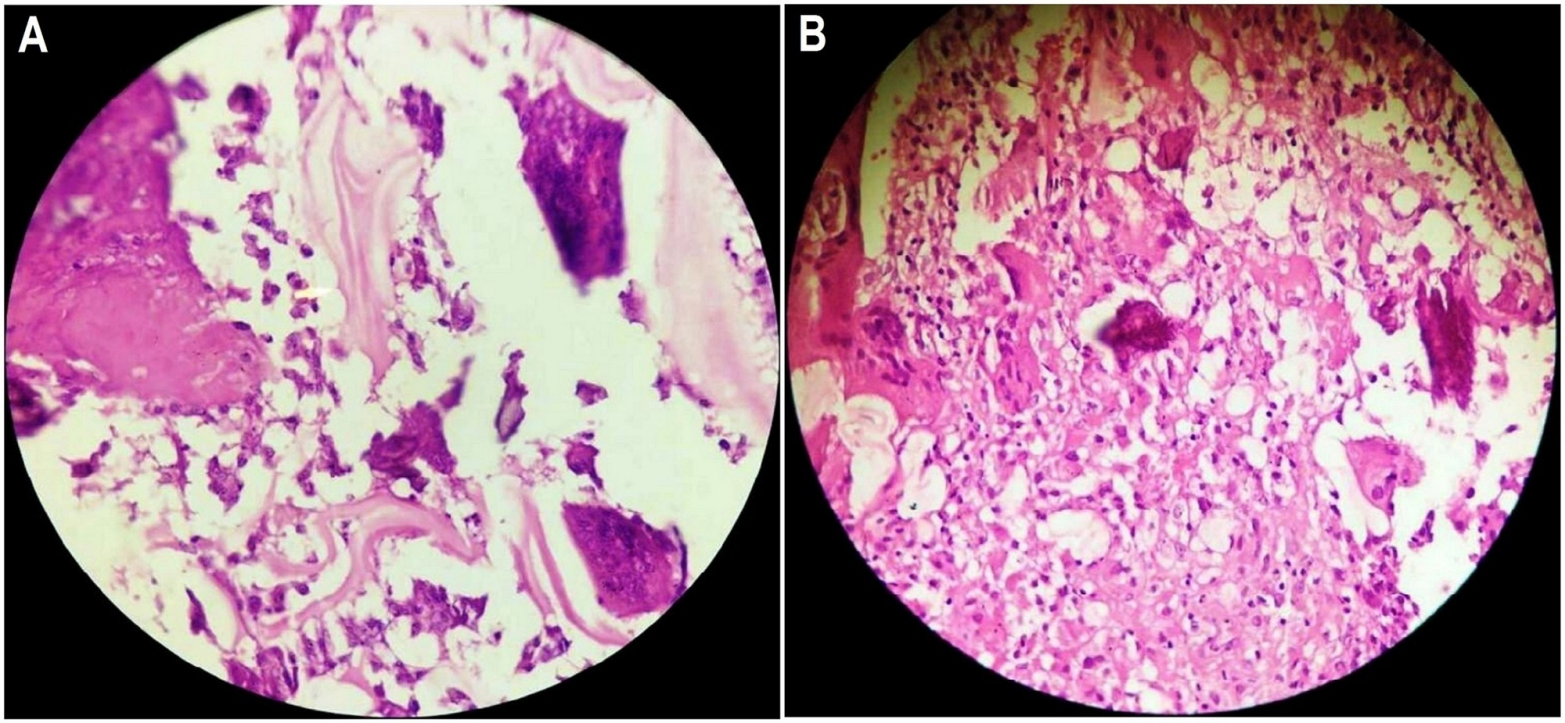
Photomicrograph of the histopathologic examination of the ilial specimen A: Three-layered acellular laminated membrane of the hydatid cyst. B: The laminated wall fragments and occasional daughter cysts are located in the center. Foreign-body giant-cell reaction is also noted (hematoxylin and eosin staining).

T-SPOT®.TB test came out negative. A whole-body bone scan showed increased uptake in the right iliac bone as well as the right sacroiliac and hip joints. Subsequently, an uneventful fine-needle aspiration biopsy was obtained from the right iliac bone. The histopathologic examination of the biopsy specimen showed the cyst wall comprised of a laminated layer and an outer layer of dense fibrovascular tissue. The fragmented tissue had laminated walls, with acute-on-chronic inflammatory infiltrates having an exuberant giant-cell reaction (Figure 3).

Differential diagnosis

On the basis of the investigative findings, tuberculosis, osteomyelitis, malignancy, aneurysm, and metastatic lesions were excluded. A diagnosis of hydatid cyst with giant cell reaction of the pelvic bone was established. A full-body evaluation was negative for liver lesions and there was no evidence of concurrent multiorgan involvement.

Treatment

**Figure 4:**
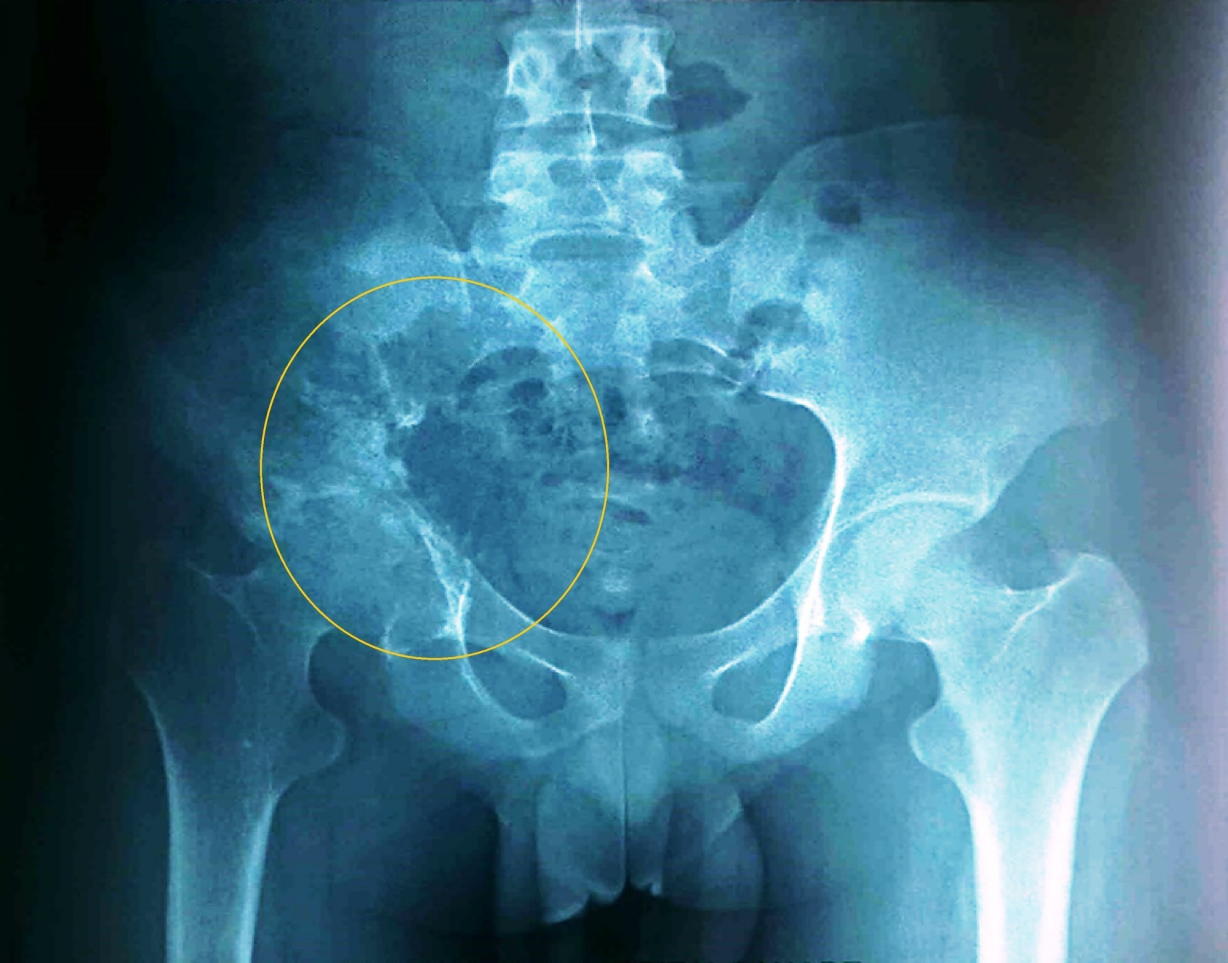
Pelvic radiograph after three months of albendazole therapy showing disease regression (anteroposterior view; circle)

The patient was initiated on oral albendazole 400 mg twice daily. After three months, repeat pelvic radiograph demonstrated a relative regression of the disease (Figure 4).

**Figure 5:**
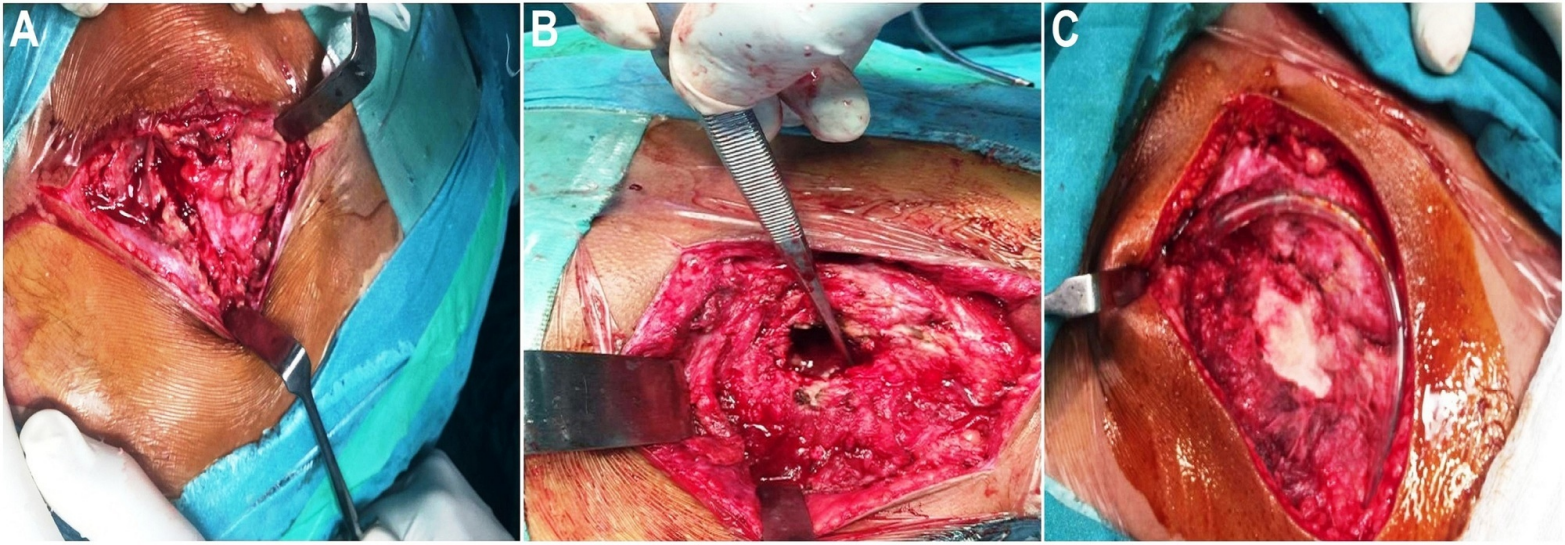
Intraoperative imaging A: The cystic area was found extending into the right iliac bone. B: A gap was noted in the iliac bone after cyst-wall excision along with curettage of the involved area. C: Debridement was performed and bone cement was placed after the cyst removal. A drain was placed according to the protocol.

Repeat whole-body bone scan showed relatively reduced uptake of radionucleotide in the right sacroiliac and hip joints and iliac bone, consistent with a slow decrease in the size of the hydatid cyst. The disease relatively regressed on the radiological scans but the clinical symptoms of the patient worsened. Thereafter, surgical intervention was undertaken. The procedure revealed a 5.5-cm cystic area in the ileum with an extension into the ischium, sacroiliac joint, and adjacent soft tissues. The hydatid cyst was carefully aspirated and the cyst wall was excised along with the curettage of the involved area. Adjacent soft tissues and sacroiliac joint were extensively debrided and the area was washed with 20% NaCl solution. A sponge soaked in hypertonic saline was placed for three minutes. The bone gap was filled with bone cement and all layers were closed in reverse order after placing a drain (Figure 5).

**Figure 6:**
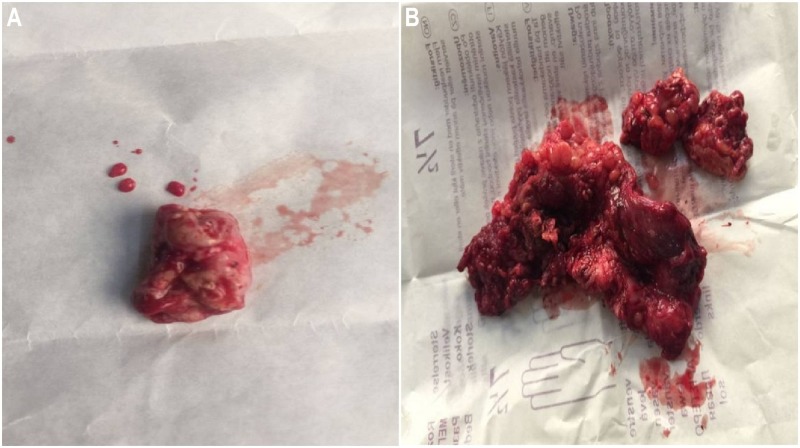
Gross morphology of the post-resection specimens A: A multilobular ovoid cystic mass. B: The soft-tissue extension of the hydatid cyst of the pelvic bones.

Particular care was taken to avoid spillage of the cystic fluid. The gross morphology of the cystic lesion resembled a multilobulated ovoid mass. Similarly, extensions of the cyst were also grossly examined and sent for histopathologic examination (Figure 6).

The results of the histological analysis of the resected specimen validated the prior biopsy findings.

Outcome and follow-up

**Figure 7:**
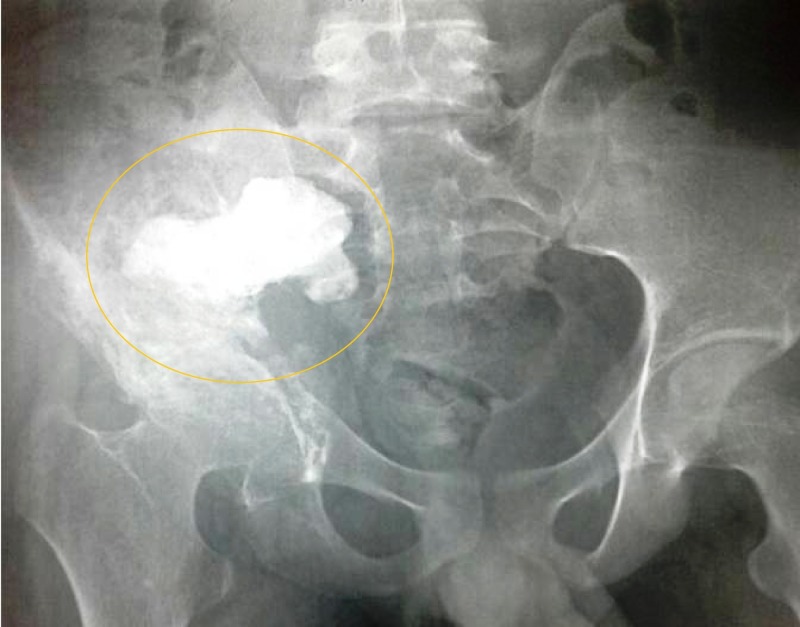
Post-procedure pelvic radiograph showing bone cement in place in the right ilium and sacroiliac joint (anteroposterior view; circle)

The post-procedure recovery was uneventful and he was discharged from the hospital in a stable condition. At the one-month follow-up visit, pelvic radiograph showed bone cement in place, with no sign of recurrence of the disease (Figure 7).

The patient is now on oral albendazole therapy with a cycle of one month with two weeks gap and his liver functions are periodically monitored. He continues to do well and is advised for a regular monthly follow-up in the outpatient department.

## Discussion

Hydatid disease is an uncommon but serious parasitic zoonosis. It is predominantly caused by a tapeworm, *E. granulosus*. This disorder is particularly prevalent in South America, the Middle East, Sub-Saharan Africa, and Central Asia [1]. A relatively higher prevalence has been noted in the rural populations that is mainly attributable to the slaughtering of animals in these areas. Several public health programmes have been initiated for the worldwide elimination of hydatidosis. However, eradication targets have not been completely achieved thus far. With regard to the body system involvement, the most commonly affected organs are the liver and the lungs due to their high vascularity [2]. The uncommon sites for cystic echinococcosis include the heart, central nervous system, and bones [3]. Cystic hydatidosis of bone is relatively a rare clinical entity, with a reported incidence of 0.5% to 4% of all patients with echinococcosis [4]. In bone disease, the vertebral column and long bones of the lower limbs are affected in over 75% of the cases. Pelvic bone hydatidosis has been described in around one-fourth of all cases of osseous hydatidosis [5].

The life cycle of *E. granulosus* has a carnivorous animal as a definitive host and an intermediate host such as sheep, cattle and other herbivores. Humans are incidental hosts due to ingestion of the parasitic eggs from contaminated food or water. Ingested eggs hatch in the small intestine and give birth to the scolices that penetrate into the intestinal mucosa and disseminate through blood or lymphatics. They migrate to different visceral sites where they are filtered out. After a few days into the body organ, a fluid-filled cyst begins to develop from scolex. It encircles itself with multiple highly-resistant layers and transforms into the hydatid cyst. Bone hydatid cyst acts as a local malignant tumor. The mechanism of invasion in patients with bone involvement is different compared to other organs as pericyst formation does not take place, enabling aggressive proliferation and spread in the bone disease. It grows in directions of least resistance, especially along the bone canals [6]. The parasite replaces the bone tissue between trabeculae with slowly growing clusters of parasitic vesicles until they reach the cortical lining. Parasitic cysts then continue to expand uniformly into the surrounding connective tissue, with cartilage and intervertebral discs offering relatively less resistance to invasion [7].

The initial clinical presentation in these patients is significantly delayed. Most *Echinococcus* infections are acquired in childhood but usually become symptomatic in late adulthood due to extremely slow progression of the disease. Therefore, the clinical presentation is usually determined by the size of the cyst and the involved organ system. In a vast majority of cases, bone cysts are asymptomatic until a pathological fracture develops [8]. In cases of osseous hydatidosis of the pelvic bone, there is no specific set of symptoms. Common presenting complaints are related to localized pain, swelling, local pressure symptoms, chronic sinus formation or claudication [9]. In published medical literature, lumbosacral neural compression leading to sciatica has also been described as the first clinical symptom of pelvic hydatidosis [10]. In these patients, physical examination may be unremarkable for abnormalities, but clinicians should remain vigilant for findings like variations in the limb symmetry, local infectious focus, fistula formation, or vertebral deformation.

Pelvic bone hydatidosis frequently poses a significant diagnostic challenge. The vague clinical features and nonspecific radiological appearance contribute to the misdiagnoses. Based on these factors, early identification of pelvic bone hydatidosis is fairly uncommon. In order to detect this abnormality using plain radiography, it is particularly important to look for the typical “waffle appearance’’ of the cystic or irregular osteolytic lesions [11]. Computed tomography (CT) scan may depict nonspecific findings in these cases. Nevertheless, the typical Echinococcal cyst assumes a round or ovoid appearance with a “double-layered arcuate calcifications” [11]. MRI is by far the best imaging modality in diagnosing osseous hydatidosis. It shows typical characteristics of the hydatid cyst with details like layers of its wall, the locularity, and the spatial relationship to the surrounding organs [12].

Serological testing is another emerging diagnostic investigation for hydatid disease. It usually involves the screening tests, including enzyme-linked immunosorbent assay (ELISA) and dot-immunogold filtration assay (DIGFA). It is notable that the overall sensitivity of DIGFA test for human cystic echinococcosis is around 80% to 90%. Major advantages of the serologic testing include convenience and timely provision of initial diagnosis [13]. A whole-body scan is usually performed to rule out the concomitant involvement of other organ systems. In these patients, major differential diagnoses such as tuberculosis, osteomyelitis, cancer, vascular aneurysms, and metastatic lesions should also be ruled out on the standard set of investigations. Routine blood tests may reveal nonspecific inflammatory trends such as eosinophilia, deranged liver function tests, and raised CRP and erythrocyte sedimentation rate (ESR) [14]. Pathologic examination of the bony lesion after surgical excision is the gold standard for definitive diagnosis. It reveals hydatid disease of the bone consisting of osseous tissue and laminated membranes of the cyst [15].

Surgical intervention coupled with antihelminthic chemotherapy is the therapeutic modality of choice [4,16]. Chemotherapy using mebendazole or albendazole as only treatment is not adequate in most patients. However, it can be employed as neoadjuvant therapy to shrink the cyst load before surgery and/or as adjuvant therapy to decrease the recurrence risk [16]. Simple debridement or drainage of the cyst using 20% normal saline is one of the surgical options that has frequently been used in the past. However, a major drawback of this technique is incomplete removal, resulting in early recurrence and dissemination of the cystic lesions. Bone cement filling is one of the proven modalities to lower recurrence rate after surgical excision. Heat generated inside the cemented filling raises the local temperature, causing necrosis of the daughter cysts. It has been demonstrated that the recurrence considerably decreased in patients who had been treated with bone cement technique, denoting an excellent long-term prognosis [17].

Complete excision and large bone grafting, total hip replacement, femoropubic arthrodesis, megaprosthetic replacement, osteosynthesis, and hemipelvectomy are included among other surgical modalities for the treatment of hydatid disease of the pelvic bone [17]. Pelvic hydatidosis is relatively difficult to diagnose at early stages because of its surreptitious progression. The concurrent involvement of the sacroiliac and hip joints makes it essentially impossible to eradicate. Despite appropriate management, a significantly high recurrence rate (8% to 22%) of pelvic hydatid disease within the first two years confers a poor prognosis. All these factors contribute to the debilitating nature of this clinicopathologic entity. Early and aggressive treatment using complete surgical excision coupled with appropriate adjuvant and/or neoadjuvant chemotherapy is the key to improve the prognosis and decrease the recurrence risk. Radiotherapy is also an effective adjunct to the surgery, especially in patients with contraindication to medical therapy [17].

The major purpose of follow-up after a therapeutic intervention is early detection of recurrence and assessment of possible complications or sequels. In patients with osseous hydatidosis, the follow-up duration must be lifelong to ensure no recurrence occurs. Although no clear guidelines are available, surveillance of recurrence should combine clinical and radiological evaluations [18]. Serologic testing has not been considered as completely reliable to determine cure or relapse after intervention as it requires several years after surgical resection to become negative. The pain, disability, and neurological problems can indicate recurrence in pelvic bone hydatidosis and should be investigated with new radiological investigations. A comparison with old imaging may help to pinpoint the culprit new cystic lesion. Furthermore, these patients may also experience the sequels of surgical treatment such as persistent pain, fractures, or gait abnormalities. Physicians should remain vigilant for other possible problems like anaphylactic reaction due to incomplete closure of cyst cavity, surgical site infection, and spontaneous superinfections [18].

## Conclusions

Hydatid disease remains a public health problem. Pelvic bone hydatidosis is rare, but it should be considered in the differential diagnosis of cystic bone lesions. This disease frequently presents a diagnostic dilemma due to nonspecific and delayed clinical presentation. Although radiological investigations often detect hydatidosis, a definitive diagnosis can only be established based on consistent histopathologic findings. The therapeutic strategy of choice is a combination of chemotherapy and surgical intervention. In these patients, debridement and excisional arthroplasty give a good functional outcome. Pelvic bone hydatidosis remains a difficult-to-treat disease as incomplete resection of the cystic lesions culminates in recurrence. This article emphasizes early diagnosis of the hydatid disease of the pelvic bone followed by appropriate management in order to salvage the bone and minimize the complications. 
